# The combination of CXCL9, CXCL10 and CXCL11 levels during primary HIV infection predicts HIV disease progression

**DOI:** 10.1186/s12967-019-02172-3

**Published:** 2019-12-13

**Authors:** Xiaowan Yin, Zhuo Wang, Tong Wu, Meichen Ma, Zining Zhang, Zhenxing Chu, Qinghai Hu, Haibo Ding, Xiaoxu Han, Junjie Xu, Hong Shang, Yongjun Jiang

**Affiliations:** 1 NHC Key Laboratory of AIDS Immunology (China Medical University), Department of Laboratory Medicine, The First Affiliated Hospital of China Medical University, Shenyang, 110001 Liaoning China; 2 Department of Clinical Laboratory, The Second Affiliated Hospital of Soochow University, Suzhou, 215004 Jiangsu China; 3 National Clinical Research Center for Laboratory Medicine, The First Affiliated Hospital of China Medical University, Shenyang, 110001 China

**Keywords:** CXCL9/MIG, CXCL10/IP-10, CXCL11/I-TAC, PHI, Disease progression

## Abstract

**Background:**

Chemokines are small chemotactic cytokines involved in inflammation, cell migration, and immune regulation in both physiological and pathological contexts. Here, we investigated the profile of chemokines during primary HIV infection (PHI).

**Methods:**

Fifty-four participants with blood samples before and during HIV infection and clinical information available were selected from an HIV-negative man who have sex with men (MSM) prospective cohort. Thirty chemokines and 10 cytokines were measured pre- and post-HIV infection in the same individuals using a Bio-Plex Pro™ Human Chemokine Panel.

**Results:**

Levels of 18 chemokines/cytokines changed significantly during PHI relative to pre-HIV infection levels; 14 were up-regulated and 4 down-regulated. Among them, CXCL9, CXCL10, and CXCL11 were the most prominently raised. Levels of CXCL9 and CXCL10 were much higher in the high-set point group (log viral load (lgVL) ≥ 4.5) than those in the low-set point group (lgVL < 4.5) and levels of CXCL9, CXCL10, and CXCL11 were higher in the low-CD4^+^ T-cell count group (CD4^+^ T-cell count ≥ 500). A formula to predict HIV disease progression using a combination panel comprising CXCL9, CXCL10, and CXCL11 was developed, where risk score = 0.007 × CXCL9 + 0.004 × CXCL10 − 0.033 × CXCL11 − 1.724, with risk score values higher than the cutoff threshold (0.5211) indicating more rapid HIV disease progression.

**Conclusions:**

A panel of plasma CXCL9, CXCL10, and CXCL11 measured during primary HIV-1 infection could predict long-term HIV disease prognosis in an MSM group and has potential as a novel biomarker in the clinic.

## Background

Chemokines are small proteins and the largest subfamily of cytokines that act on G protein-coupled receptors (GPCRs) with seven transmembrane domains [1]. Chemokines are classified into four subfamilies according to their structural characteristics: CXC, CC, CX3C, and XC [2]. To date, 17 CXC-chemokines, 28 CC chemokines, 2 XC chemokines, 1 CX3C chemokine, and approximately 20 chemokine receptors have been identified [3, 4]. Plasma chemokines are primarily secreted by monocytes, T cells, dendritic cells (DCs), and some epithelia [3]. Chemokines can attract immunocytes to areas of inflammation, infection, and tissue damage [5], and are critical in the development and differentiation of immune cells. Additionally, chemokines contribute to the pathogenesis of multiple diseases, including autoimmune disorders, hypersensitive reactions, cancers, and viral infections [1, 6–8]. A chemokine panel was used to screen plasma samples from patients with colorectal cancer, and five chemokines and four cytokines identified as associated with increased total mortality, among which TNF-α and CCL24 were exclusively associated with colorectal cancer-specific mortality [9]. Further, Weisheng et al. [10] identified a 14-biomarker panel (chemokines and cytokines) for detecting endometriosis. Hence, chemokine panels can be valuable for disease diagnosis and/or progression prediction.

Primary HIV infection (PHI) refers to the early period of HIV infection (approximately the first 12 weeks after infection) [11], during which the virus disseminates from the original infection site to different tissues and organs. Subsequently, numerous events occur, including establishment of a viral set-point, which determines subsequent HIV progression [12–16]. Different immune cell subsets are activated in response to HIV and secrete large quantities of cytokines and chemokines potentially associated with disease progression, opportunistic infection, and increased mortality [17–20].

Investigators have focused on the immunological role of chemokines in early HIV infection. During acute simian immunodeficiency virus (SIV) infection, levels of MCP-1, MIP-1α, and MIP-1β can distinguish progressive and non-progressive SIV infection in *Chlorocebus sabaeus* [21]. Further, seven human plasma chemokines were assessed, and fold-change in CXCL10 (HIV^+^ vs. HIV^−^ plasma level) was significantly higher in HIV rapid progressors, with CXCL10 level during PHI negatively correlated with CD4^+^ T-cell counts at the 4-month-infection point [22]. Additionally, 15 cytokines and 1 chemokine (CXCL10) are present at higher levels in rapid relative to slow disease progressors during acute HIV-1 infection [23]. Furthermore, the combination of IL-12p40, IL-12p70, IFN-γ, IL-7, and IL-15, but not chemokines, could predict HIV disease progression in women with acute HIV-1 infection [24]. Overall, cytokine levels during HIV infection have been studied; however, only 6–8 chemokines were generally assessed, hence the magnitude of alterations in the majority of chemokine profiles during PHI remain unknown. Further, studies usually compare concentrations of chemokines in samples from HIV-positive and healthy HIV-negative individuals, and high-within-person-variability can result in measurement errors. Changes in chemokine profiles pre- and post-HIV infection during PHI in the same individual may more accurately represent disease conditions.

Here, we used 108 plasma samples collected from 54 patients at two sampling points (pre- and post-PHI) to determine alterations in profiles of 30 chemokines and 10 cytokines between the two sampling points. Furthermore, we analyzed the relationship between chemokine concentrations and disease progression. Finally, we developed the combination of CXCL9, CXCL10, and CXCL11 levels during PHI as a biomarker to predict HIV disease progression.

## Methods

### Study participants

We set up a prospective open cohort study in the Key Laboratory of AIDS Immunology of the National Health and Family Planning Commission [The First Affiliated Hospital of China Medical University (CMU)], which to date includes > 2000 men who had sex with men (MSM) high-risk study participants, who were HIV-negative when they were enrolled, and all of whom have been screened for HIV infection every 1–3 months. Among newly-diagnosed participants, 54 participants with available clinical information and blood samples from two time points; pre-HIV infection (HIV seronegative and HIV RNA-negative) and post-HIV infection (HIV seropositive or HIV RNA-positive), were selected; the estimated time of HIV infection ranged from 13 to 155 days (Table 1). The estimated time of infection was defined as previously described [25]. Briefly: (i) by referring to Fiebig stage [26]; (ii) if the patient could clearly recall the time of high-risk exposure, that time point was the estimated infection time [25]; (iii) the midpoint between the last time point of HIV antibody negative test and the first HIV antibody positive test was the estimated infection time [25]. On collection, all plasma samples were immediately stored at − 80 °C until use. After diagnosis with HIV infection, the 54 participants were followed up for an average of 1745 days (range from 7 to 3431 days). All clinical study protocols were approved by the Ethics Review Committee of The First Affiliated Hospital of China Medical University, Shenyang, P. R. China, and the study was conducted according to the principles of the Declaration of Helsinki ([2018] 2015-140-5).

**Table 1:** Patient demographic and clinical characteristics

Characteristic	Pre-HIV infection (HIV^−^)	Post-HIV infection (HIV^+^)
***Sociodemographic variables***		
N	54	
Sex	Male (54/54)	
Age, years [median (IQR)]^a^	32.5 (28, 41)	
< 20	0	
20–30	19	
30–40	20	
40–50	10	
> 50	5	
Unknown	4	
Marital status [*n*/total (%)]		
Unmarried	45 (83%)	
Married	6 (11%)	
Divorced	3 (6%)	
MSM	54/54	
Chinese	54/54	
Ethnicity	Han (45/54)	
	Man (7/54)	
	Other (2/54)	
***Clinical***		
HBV Ag	N/A	4/54
HCV Ab	N/A	1/54
TPPA	N/A	26/54
Fiebig staging		
I–II	–	14
III–IV	–	16
V–VI	–	24
Estimated infection day (days) [median (IQR)]^a^	− 91 (− 141, − 56)	31 (25, 49)
Methods for estimating infection day (*N*)		
i. Fiebig stage	N/A	6
ii. Remembered time of high-risk exposure	N/A	24
iii. Laboratory HIV antibody detection	N/A	24
Follow-up (days) [median (IQR)]^a^	N/A	1719 (1142, 2625)
***CD4***^***+***^***T-cell count (cells/μL) [median (IQR)]***^a^		
Chemokine detection sampling point	N/A	424 (328, 531)
Set-point	N/A	488 (390, 634)
One-year-infection point^b^	N/A	412 (328, 555)
***Viral load (copies/mL) [median (IQR)]***^a^		
Chemokines detection sampling point	N/A	78,500 (11,370, 753,000)
Set-point	N/A	24,900 (8450, 83,050)
One-year-infection point^b^	N/A	22,887 (8482.5, 36,600)
Viral subtype [N/total]	–	CRF-01AE (53/54)
		CRF-01AE/BC (1/54)

*N/A* not applicable, *MSM* men who have sex with men

^a^Data presented as median (IQR, interquartile range)

^b^Antiretroviral therapy-naïve patient at 1-year-infection point

### Detection of CD4^+^ T-cell count and HIV viral load (VL)

The FACSCalibur (BD, Franklin Lakes, New Jersey, USA) flow cytometer were used to measured absolute blood CD4^+^ T-cell counts (cells/μL). The levels of plasma HIV-1 RNA (copies/mL) were detected by the COBAS Ampliprep/COBAS TaqMan 48 Analyzer (Roche Diagnostics, Branchburg, New Jersey, USA).

### Plasma chemokine/cytokine detection

Plasma chemokine/cytokine levels were determined using a Bio-Plex Pro™ Human Chemokine Panel 40-Plex (BIO-RAD), including CXCL1/Gro-α, CXCL2/Gro-β, CXCL5/ENA-78, CXCL6/GCP-2, CXCL8/IL-8, CXCL9/MIG, CXCL10/IP-10, CXCL11/I-TAC, CXCL12/SDF-1A+β, CXCL13/BCA-1, CXCL16/SCYB16, CCL1/I-309, CCL2/MCP-1, CCL3/MIP-1α, CCL7/MCP-3, CCL8/MCP-2, CCL11/Eotaxin, CCL13/MCP-4, CCL15/MIP-1δ, CCL17/TARC, CCL19/MIP-3β, CCL20/MIP-3α, CCL21/6Ckine, CCL22/MDC, CCL23/MPIF-1, CCL24/Eotaxin-2, CCL25/TECK, CCL26/Eotaxin-3, CCL27/CTACK, CX3CL1/Fractalkine, GM-CSF, MIF, TNF-α, IFN-γ, IL-1β, IL-2, IL-4, IL-6, IL-10, and IL-16. Chemokine/cytokine standards supplied by the manufacturer were detected in duplicate and run on each plate. All data were acquired using a Bio-Plex 200 System (BIO-RAD). Chemokine/cytokine levels below/above the minimum/maximum thresholds of detection are reported as the minimum/maximum threshold values for each chemokine/cytokine.

### Statistical analysis

Statistical analyses were performed using GraphPad Prism version 6.0 and SPSS Statistics 20.0. All tests were two-tailed and *p* values < 0.05 were considered significant. The nonparametric Wilcoxon matched pairs test was used to evaluate differences in chemokine/cytokine levels from the same individual at different time points (i.e., pre-and post-HIV infection). The nonparametric Mann–Whitney U test was used to compare between-group distributions. Spearman correlation analysis was used to estimate correlation. Kaplan–Meier curves were used to estimate the time to an outcome (CD4^+^ T-cell counts ≤ 500 or VL > 10^4^ copies/mL). The predictive value of the combination of CXCL9, CXCL10, and CXCL11 levels for HIV disease progression was estimated using receiver operating characteristic (ROC) curves. Predictive values were expressed as the area under the curve (AUC). For ROC analysis of CXCL9, CXCL10, and CXCL11 combinations, *p* (probability of a patient sample) was calculated for inclusion in the ROC analysis using the formula:

$$X = logit\left( p \right) = \ln \left( {\frac{p}{1 - p}} \right) = b_{0} + b_{1} \Delta CT_{1} + b_{2} \Delta CT_{2} + b_{3} \Delta CT_{3} \ldots + b_{n} \Delta CT_{n}$$

where b_i_ terms were the ith regression coefficients by binary logistic regression, and ∆CT_i_ terms were the relative expression levels of each chemokine [24]. In this study, a CXCL9, CXCL10, and CXCL11-chemokine combination panel (X = − 1.724 + 0.007 × CXCL9 + 0.004 × CXCL10 − 0.033 × CXCL11, and *p *= e^X^/(1 + e^X^)) was used predict disease progression.

## Results

### Study participants

Fifty-four HIV-infected individuals from an MSM cohort were recruited to this study. Their basic information is presented in Table 1. Most were unmarried (76%). Median estimated infection day was 31 days and median CD4^+^ T-cell count and VL at the chemokine detection sampling point during PHI were 424 cells/μL and 78,500 copies/mL, respectively. Five patients were followed for < 120 days, 14 received antiretroviral therapy immediately after diagnosis, and set-point samples were collected from the remaining 35, with median CD4^+^ T-cell count and VL at set-points of 488 cells/μL and 24,900 copies/mL, respectively. Thirty-two HIV-infected individuals were followed for at least 1 year without antiretroviral therapy, with median CD4^+^ T-cell count and VL at 1-year-infection of 412 cells/μL and 22,887 copies/mL, respectively.

### CXCL9, CXCL10, and CXCL11 were the most prominently raised of 16 chemokines with altered levels during PHI relative to pre-HIV infection

**Fig. 1:**
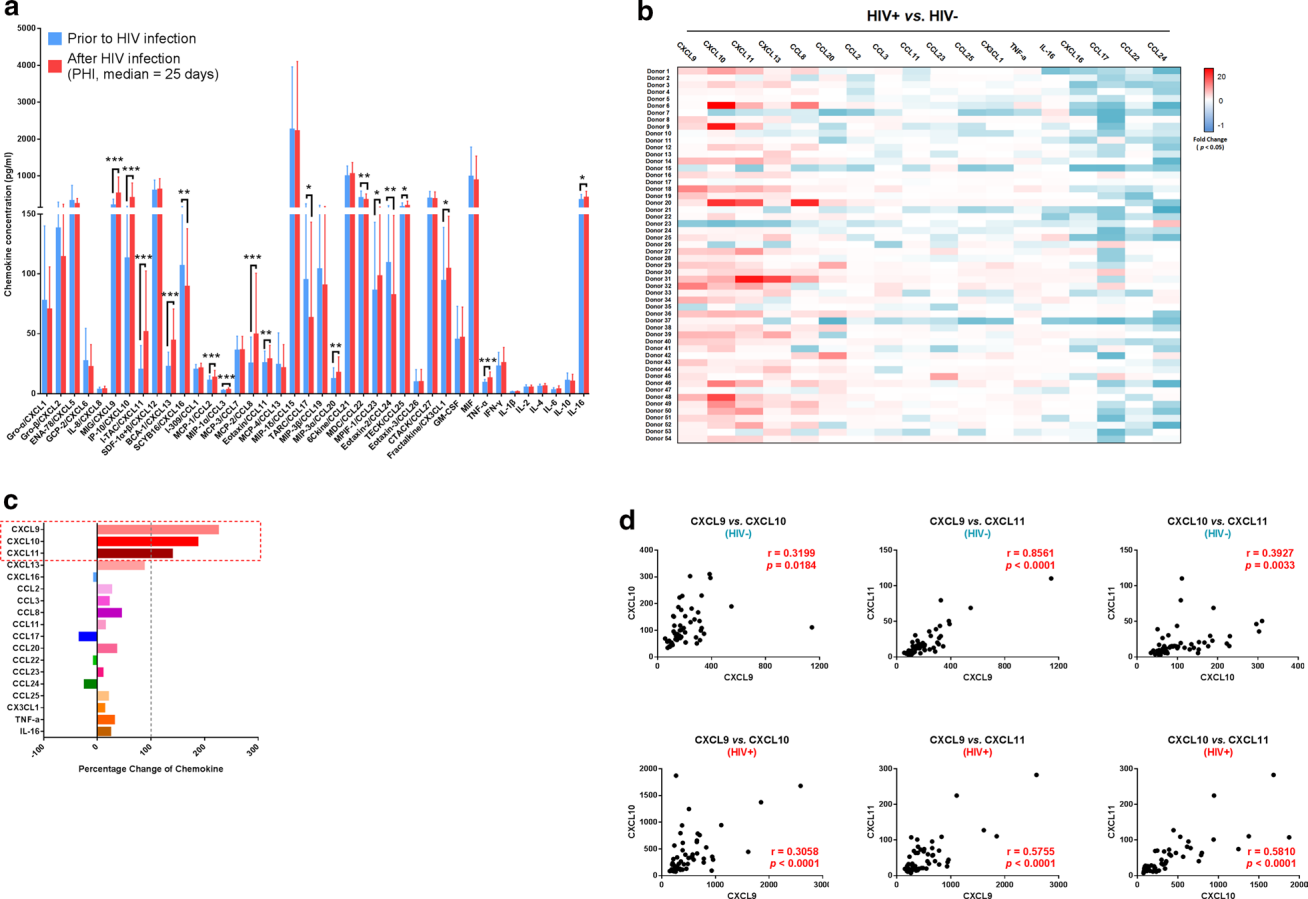
Alterations in the levels of 40 plasma chemokines/cytokines pre- and post-HIV infection. **a** Comparison of the levels of 40 chemokines/cytokines pre- and post-HIV infection in the same individuals (n = 54). Graphs represent median ± interquartile range. The nonparametric Wilcoxon matched pairs test was used for evaluation of the significance of differences between groups. **b** Heat map showing the fold-change values of 18 chemokines/cytokines with levels significantly changed in each individual during HIV infection. White, non-significant fold-change in chemokine/cytokine levels between the two time points (HIV^−^ and HIV^+^). Blue and red, significantly decreased or increased fold-change in chemokine/cytokine levels between the two time points (HIV^−^ and HIV^+^), respectively. The darker and more saturated the color, the greater the extent of the fold-change. **c** Percentage change in the 18 chemokines/cytokines with significantly altered levels: CXCL9, CXCL10, CXCL11, CXCL13, CXCL16, CCL2, CCL3, CCL8, CCL11, CCL17, CCL20, CCL22, CCL23, CCL24, CCL25, CX3CL1, TNF-α, and IL-16. **d** Correlation analysis of plasma CXCL9, CXCL10, and CXCL11 levels in HIV^−^ and HIV^+^ samples. Pearson’s correlation coefficient values between chemokines are shown. *p < 0.05, **p < 0.01, ***p < 0.001, ****p < 0.0001

To clarify the profile of chemokine changes during PHI, we compared the levels of 30 chemokines and 10 cytokines in blood plasma from the same individuals at two time points, pre and post-HIV infection. Of the 40 measured chemokines/cytokines, 18 were significantly changed in blood plasma post-HIV infection (Fig. 1a); fold-change values for each chemokine/cytokine in each individual are presented as a heat map (Fig. 1b). Moreover, 16 of the 18 significantly altered chemokines/cytokines were chemokines, among which 12 were up-regulated and four down-regulated. Only two cytokines, TNF-α and IL-16, were up-regulated during PHI. Further, comparison of percentage change values [(HIV-positive chemokine level − HIV-negative chemokine level)/HIV-negative chemokine level × 100%] among the 18 significantly altered chemokines/cytokines revealed alterations ranging from − 33.24% to 225.26%. Interestingly, among up-regulated chemokines, CXCL9, CXCL10, and CXCL11 were those with the highest percentage change: 225.26%, 187.02%, and 139.29%, respectively (Fig. 1c).

As CXCL9, CXCL10, and CXCL11 all bind to the same receptor chemokine, (C-X-C motif) receptor 3 (CXCR3), further statistical analysis was conducted to examine whether the changes in these three chemokines were interrelated. As shown in Fig. 1d, analysis of correlation between levels of any two among CXCL9, CXCL10, and CXCL11, either pre- or post-HIV infection, revealed positive correlations. Thus, the three most up-regulated chemokines in PHI may impact disease progression.

### Levels of CXCL9, CXCL10, or CXCL11 were associated with CD4^+^ T-cell count and VL

**Fig. 2:**
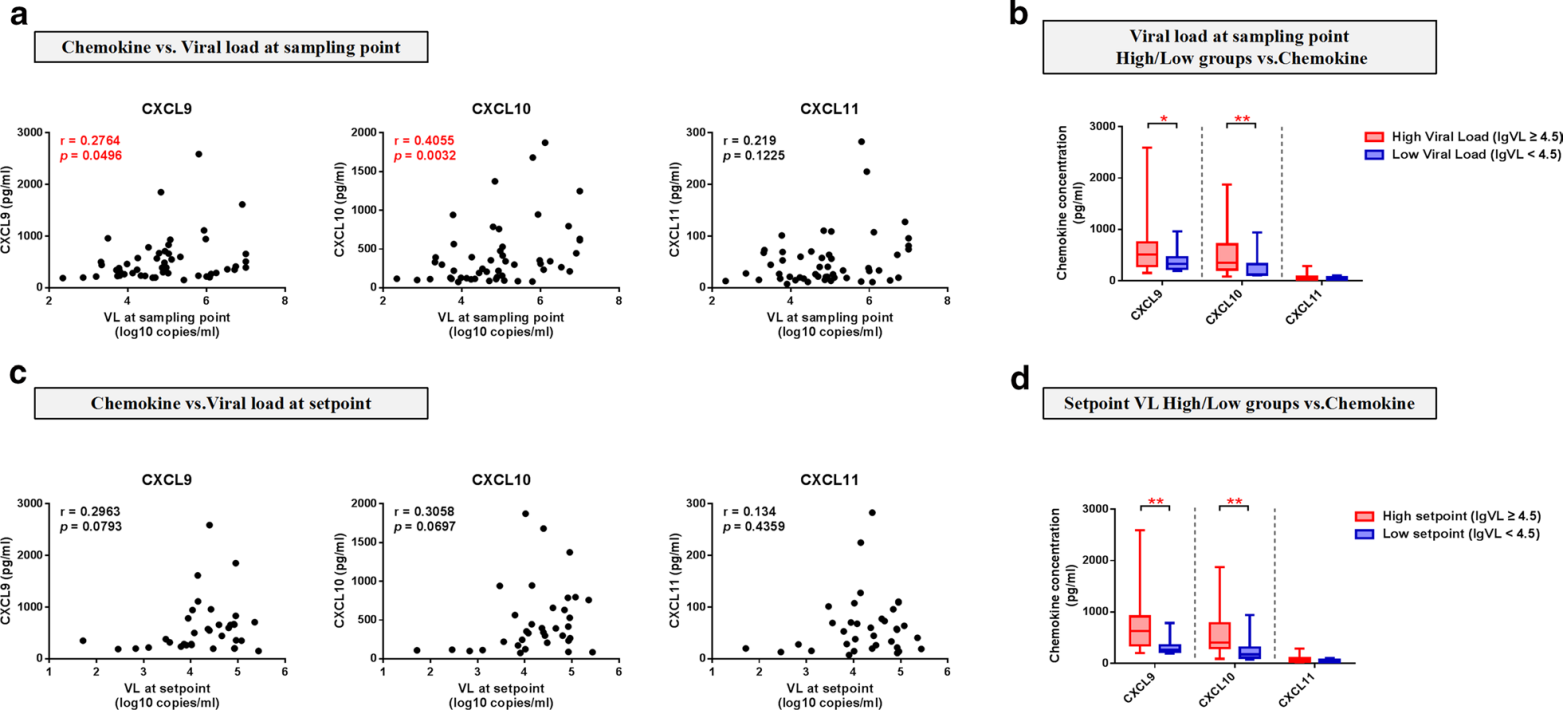
Plasma CXCL9, CXCL10, and CXCL11 levels during PHI were correlated with viral load. **a** Analysis of correlations between plasma CXCL9 (r = 0.2764, *p *= 0.0496), CXCL10 (r = 0.4055, *p *= 0.0032), and CXCL11 (r = 0.219, *p *= 0.1225) levels in HIV^+^ samples and VL at the same sampling point (n = 51). **b** Comparisons of CXCL9, CXCL10, and CXCL11 levels between VL-high (n = 33) and VL-low (n = 18) groups at the sampling point (CXCL9, *p* = 0.0381; CXCL10, *p* = 0.0079; CXCL11, *p* = 0.1161). **c** Analysis of the correlations between plasma CXCL9 (r = 0.2963, *p *= 0.0793), CXCL10 (r = 0.3058, *p *= 0.0697), and CXCL11 (r = 0.134, *p *= 0.4359) levels at the HIV^+^ point and VL at set-point (n = 35). **d** Comparisons of CXCL9, CXCL10, and CXCL11 levels between the VL-high (n = 24) and VL-low (n = 11) groups at set-point (CXCL9, *p* = 0.0035; CXCL10, *p* = 0.0052; CXCL11, *p* = 0.0896). Spearman correlation analysis was used to estimate the level of correlation. A nonparametric Mann–Whitney U test was used to compare between-group distributions; *p < 0.05, **p < 0.01, ***p < 0.001, ****p < 0.0001

To further evaluate the association of up-regulation of CXCL9, CXCL10, CXCL11 and HIV disease progression, we analyzed the correlation between plasma chemokine levels during PHI and parameters related to HIV disease progression. First, we evaluated the relationship between the three chemokines and VL measured at the same sampling time point. CXCL9 and CXCL10 were positively correlated with VL (CXCL9, r = 0.2764, *p *= 0.0496; CXCL10, r = 0.4055, *p *= 0.0032) (Fig. 2a). Further, we categorized participants into two groups, according to VL level at the sampling point, with a threshold of 4.5 (VL-high, log VL (lgVL) ≥ 4.5, n = 33; VL-low, lgVL < 4.5, n = 18), and determined whether the groups had differing chemokine levels during PHI. CXCL9 and CXCL10 levels in the VL-high group were both higher than those in the VL-low group (*p *= 0.0381 and *p *= 0.0079, respectively) (Fig. 2b). As VL set-point measured at 120 days post-HIV-infection is recognized as an index of disease progression, we next evaluated the relationship between levels of the three chemokines and VL at set-point. We detected a tendency toward correlation between CXCL9 and CXCL10 with VL at set-point; however, the relationships were not significant (CXCL9, *p *= 0.0793; CXCL10, *p *= 0.0697) (Fig. 2c). We next categorized participants into high (lgVL ≥ 4.5, n = 24) and low (lgVL < 4.5, n = 11) set-point value groups and found that, compared with the low VL set-point group, levels of CXCL9 (*p* = 0.0035) and CXCL10 (*p* = 0.0052) were elevated in the high VL set-point group (Fig. 2d).

**Fig. 3:**
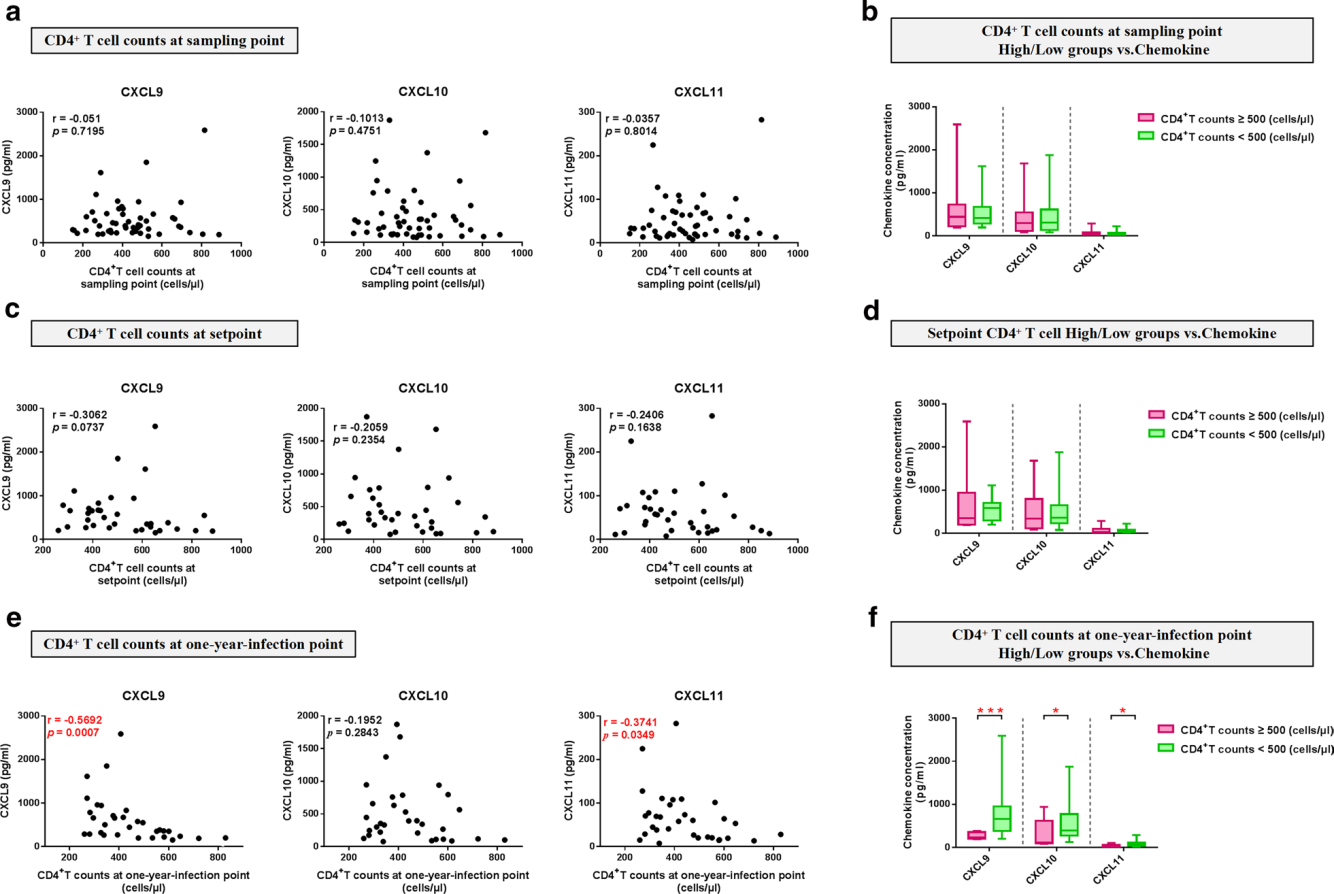
Plasma CXCL9, CXCL10 and CXCL11 levels during PHI are correlated with CD4^+^ T-cell counts. **a** Analysis of correlations between plasma CXCL9 (r = − 0.051, *p *= 0.7195), CXCL10 (r = − 0.1013, *p *= 0.4751), and CXCL11 (r = -0.0357, *p *= 0.8014) levels at the HIV^+^ point and CD4^+^ T-cell count at the same sampling point (n = 52). **b** Comparisons of CXCL9, CXCL10, and CXCL11 levels between the CD4^+^-high (n = 16) and CD4^+^-low (n = 36) groups at the sampling point (CXCL9, *p* = 0.9448; CXCL10, *p* = 0.7407; CXCL11, *p* = 0.9893). **c** Analysis of the correlations between plasma CXCL9 (r = − 0.3062, *p *= 0.0737), CXCL10 (r = − 0.2059, *p *= 0.2354), and CXCL11 (r = − 0.2406, *p *= 0.1638) levels at the HIV^+^ point and CD4^+^ T-cell count at set-point (n = 35). **d** Comparisons of CXCL9, CXCL10, and CXCL11 levels between the CD4^+^-high (n = 16) and CD4^+^-low (n = 19) groups at set-point (CXCL9, *p* = 0.1662; CXCL10, *p* = 0.5256; CXCL11, *p* = 0.3317). **e** Analysis of the correlations between plasma CXCL9 (r = − 0.5692, *p *= 0.0007), CXCL10 (r = -0.1952, *p *= 0.2843), and CXCL11 (r = − 0.3741, *p *= 0.0349) levels at the HIV^+^ point and CD4^+^ T-cell count at 1-year-infection (n = 32). **f** Comparisons of CXCL9, CXCL10, and CXCL11 levels between the CD4^+^-high (n = 10) and CD4^+^-low (n = 22) groups at 1-year-infection (CXCL9, *p* = 0.0008; CXCL10, *p* = 0.0242; CXCL11, *p* = 0.0106)

Next, we evaluated the relationship between levels of the three chemokines and another key index of HIV disease progression, CD4^+^ T-cell count. We found no significant correlation with CD4^+^ T-cell counts at either the sampling or set-points (Fig. 3a, c). Further, we analyzed the relationship between the three significantly altered chemokines and CD4^+^ T-cell count at the 1-year-infection point without antiretroviral therapy, as an approximation of HIV disease progression. CXCL9 and CXCL11 were negatively correlated with CD4^+^ T-cell count at 1-year-infection point (CXCL9, r = − 0.5692, *p *= 0.0007; CXCL11, r = − 0.3741, *p *= 0.0349) (Fig. 3e). Furthermore, participants were categorized into two groups, according to CD4 < 500 and CD4 ≥ 500; [(i) sampling point: CD4-high, n = 16, CD4-low, n = 36; (ii) set-point: CD4-high, n = 16, CD4-low, n = 19; (iii) 1-year-infection point: CD4-high, n = 10, CD4-low, n = 22)]. We found no significant difference in CXCL9, CXCL10, and CXCL11 levels between the CD4-high and CD4-low groups at either the sampling or set-points (Fig. 3b, d); however, levels of all three chemokines were higher in the CD4 < 500 group relative to those in the CD4 ≥ 500 group at the 1-year-infection point (CXCL9, *p *= 0.0008; CXCL10, *p *= 0.0242; CXCL11, *p *= 0.0106) (Fig. 3f).

Together, our results demonstrate that CXCL9, CXCL10, or CXCL11 levels during PHI are associated with multiple indices of HIV infection, and that higher levels of chemokines during PHI may indicate rapid HIV disease progression.

### Dynamic changes in CXCL9, CXCL10, and CXCL11 during PHI

**Fig. 4:**
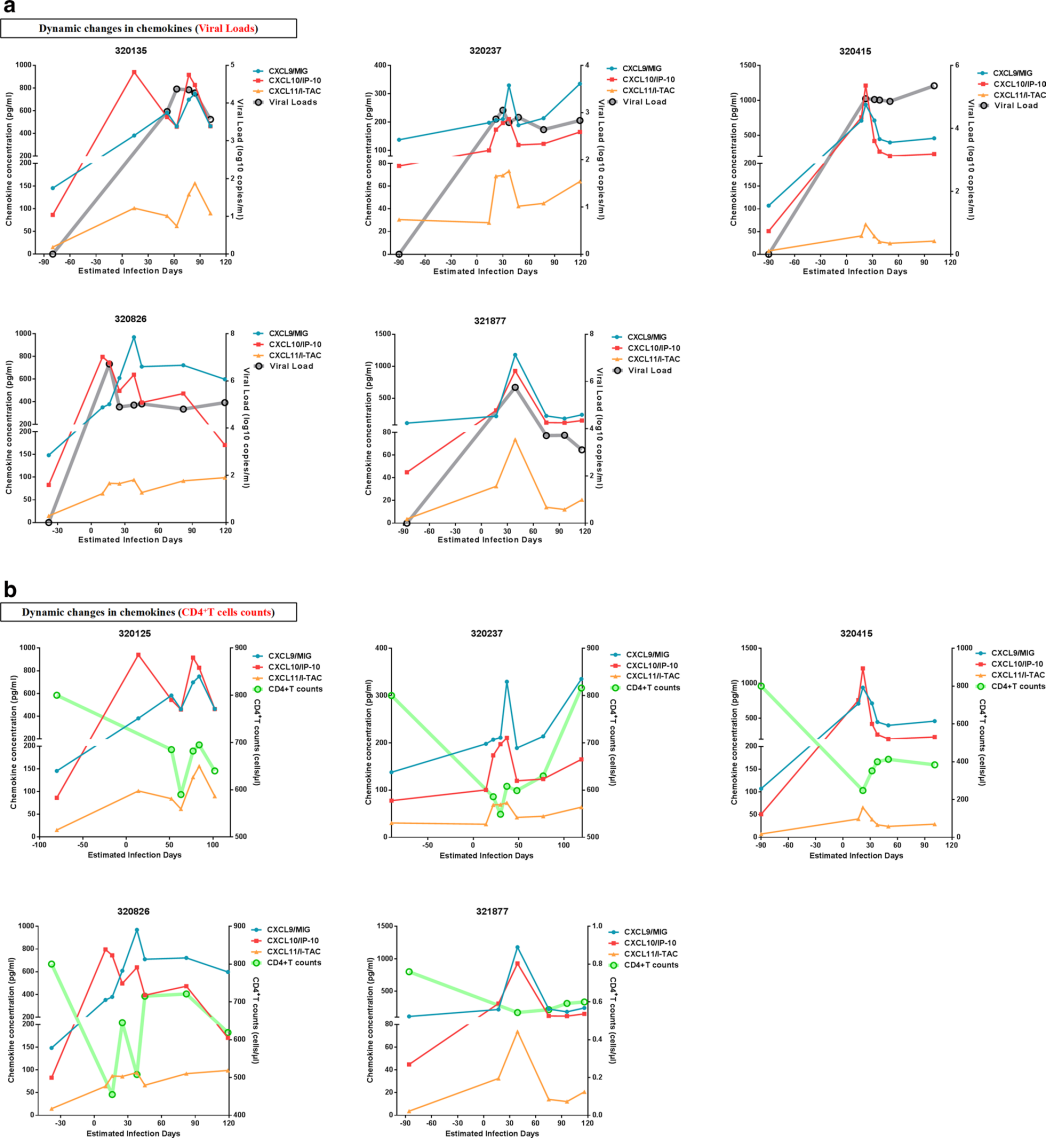
Dynamic changes in plasma CXCL9, CXCL10, and CXCL11 levels during PHI. **a** Dynamic changes in plasma CXCL9, CXCL10, CXCL11, and viral load during PHI in five participants. Blue, red, yellow, and gray lines represent CXCL9, CXCL10, CXCL11, and viral load, respectively. **b** Dynamic changes in plasma CXCL9, CXCL10, CXCL11, and CD4^+^ T-cell count during PHI in five participants. Blue, red, yellow, and green lines represent CXCL9, CXCL10, CXCL11, and CD4^+^ T-cell count, respectively

To investigate the dynamics of plasma CXCL9, CXCL10 and CXCL11 during PHI, sequential longitudinal plasma samples, collected from five individuals pre- to post-infection (6–8 sampling points/patient) were analyzed. Plasma levels of each chemokine and estimated infection time are presented in Fig. 4. All three chemokines were significantly elevated post-HIV infection, and changes in levels of the three chemokines were consistent with those of VL, with patterns of fluctuation of the three chemokines similar during PHI (Fig. 4a, b). Levels of CXCL10 in two patients peaked earlier than those of CXCL9 and CXCL11. Simultaneously, peak levels of the three chemokines were consistent with lowest CD4^+^ T-cell counts during PHI. Although we did not test pre-infection CD4^+^ T-cell counts in these participants, the mean pre-infection value was 800 cells/μL in this cohort; therefore, we set 800 cells/μL as the initial CD4^+^ T-cell count pre-HIV infection. Together, these data illustrate the dynamic features of elevations in CXCL9, CXCL10, and CXCL11 levels during HIV infection, including rapid and transient elevations in these chemokines as viremia increased and CD4^+^ T-cell counts decreased.

### The combination of CXCL9, CXCL10, and CXCL11 represents a key biomarker for predicting HIV disease progression

**Fig. 5:**
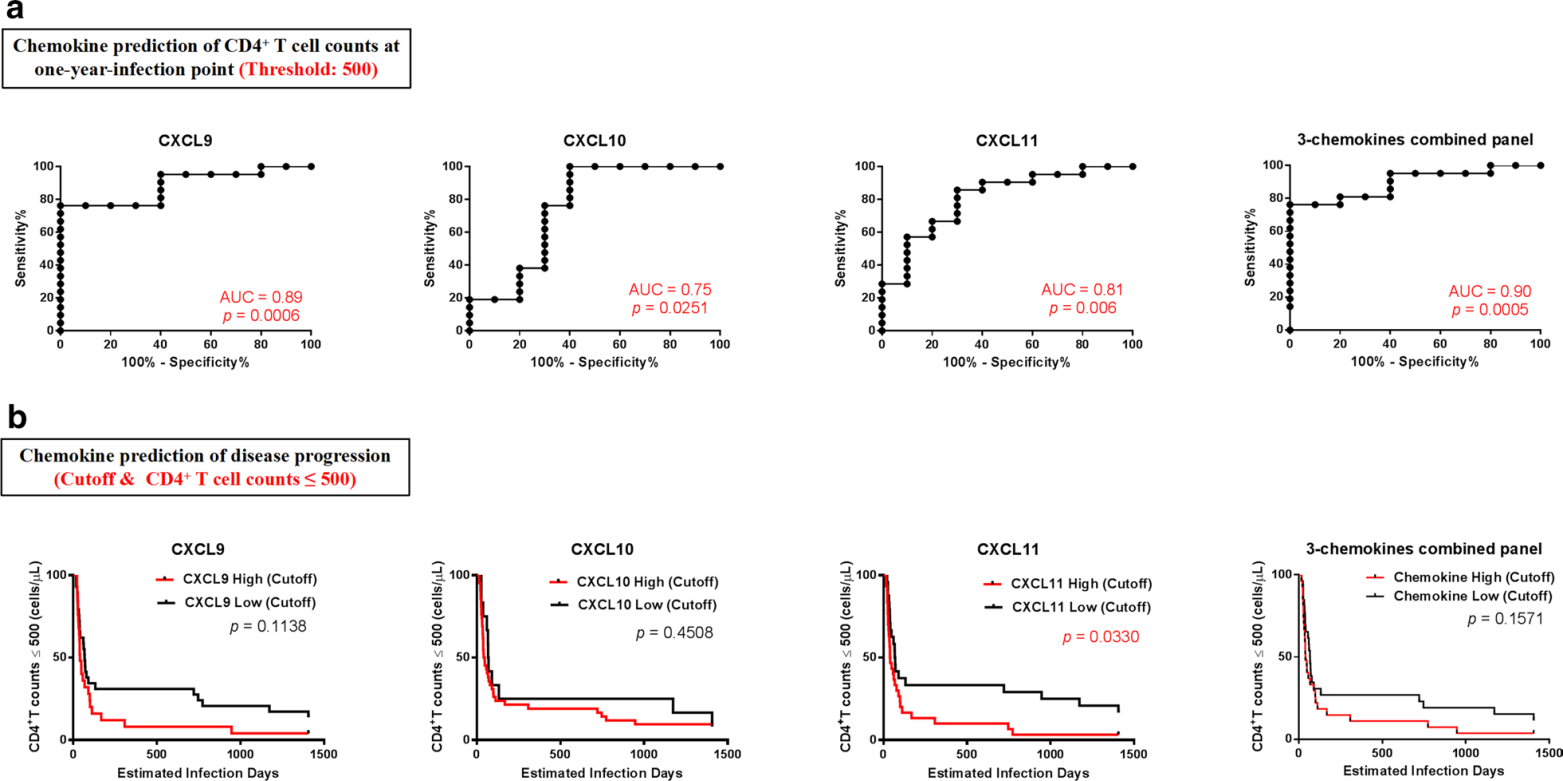
Value of CXCL9, CXCL10, and CXCL11 levels for predicting HIV CD4^+^ T-cell count. **a** ROC curves for CXCL9, CXCL10, CXCL11, and a combination panel of three chemokines, in predicting CD4^+^ T-cell count at 1-year-infection. AUC values were used to evaluate CD4^+^ T-cell count at 1-year-infection and the point with the maximum sum of sensitivity plus specificity was defined as the cutoff value. **b** Kaplan–Meier curves for CXCL9, CXCL10, CXCL11, and the combination panel of three chemokines. Patients were divided into high expressers (above the cutoff value) and low expressers (below the cutoff value), according to the cutoff value for chemokine expression. CD4^+^ T-cell count reaching ≤ 500 cells/μL was considered as the end point for follow-up. *p* values for curves provided on graphs

As CXCL9, CXCL10, and CXCL11 were associated with HIV disease progression parameters, we next assessed whether their levels during PHI are a potential biomarker for prediction of HIV disease progression. We investigated whether chemokine levels in PHI could predict CD4^+^ T-cell count at 1-year-infection, using a threshold of 500 cells/μL for distinguishing CD4^+^ T-cell count groups. ROC curve analysis showed that the predictive value of CXCL9 was 89% (*p* = 0.0006), CXCL10 75% (*p* = 0.0251), and CXCL11 81% (*p* = 0.006), and the three-chemokine combined panel had a predictive value of 90% (*p* = 0.0005). Using these ROC curves, we defined the cutoff values for CXCL9, CXCL10, CXCL11, and the three-chemokine combined panel as 412 pg/mL, 120.9 pg/mL, 28.24 pg/mL, and 0.6841, respectively (Fig. 5a). Furthermore, we used these cutoff values to verify the predictive value of chemokine levels during PHI for disease progression in these participants. Participants were followed up for 1500 days, and outcomes defined as CD4^+^ T-cell count < 500 cells/μL. Kaplan–Meier curves showed that disease progression in the CXCL11-high group during PHI was more rapid than that in the CXCL11-low group (*p *= 0.033); however, there was no significant difference in disease progression between the CXCL9 (*p *= 0.1138), CXCL10 (*p *= 0.4508), or the three-chemokine combined panel (*p *= 0.1571)-high and -low groups (Fig. 5b).

**Fig. 6:**
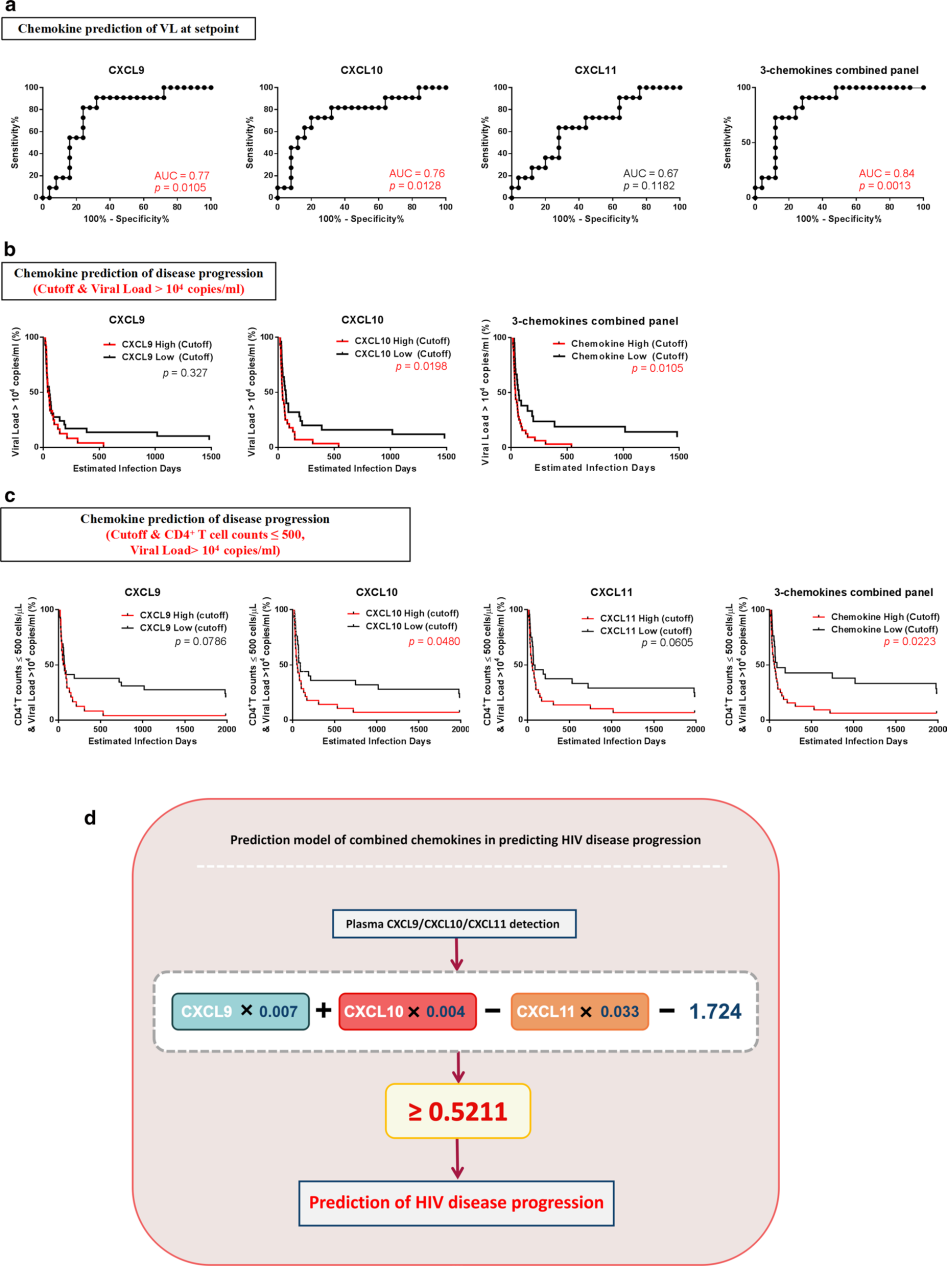
Value of CXCL9, CXCL10, and CXCL11 for predicting HIV viral load and disease progression. **a** ROC curves for CXCL9, CXCL10, CXCL11, and the combination panel of three chemokines to evaluate their use for predicting viral load at set-point. AUC was used to evaluate viral load at set-point and the point with the maximum sum of sensitivity plus specificity was defined as the cutoff value. **b** Kaplan–Meier curves for CXCL9, CXCL10, CXCL11, and the combination panel of three chemokines. According to the cutoff value for chemokine expression levels, patients were divided into high expressers (above the cutoff value) and low expressers (below the cutoff value). Viral load reaching > 10^4^ copies/mL was considered the end point of follow-up. *p* values for curves provided on graphs. **c** Kaplan–Meier curves for CXCL9, CXCL10, CXCL11, and the combination panel of three chemokines. According to the cutoff value of chemokine expression, patients were divided into high expressers (above the cutoff value) and low expressers (below the cutoff value). Viral load reaching > 10^4^ copies/mL and CD4^+^ T-cell count reaching ≤ 500 cells/μL was considered the end point for follow-up. *p* values for curves provided on graphs. **d** Schematic and formula of the combined-chemokine-predictive model for HIV disease progression

We then analyzed the predictive value of chemokines for VL set-points. Using ROC analysis, the predictive accuracy for CXCL9 (*p* = 0.0105) and CXCL10 (*p* = 0.0128) were 77% and 76%, respectively, based on AUC values for rapid disease progression (Fig. 6a). Next, we analyzed the predictive value of the combined panel of CXCL9, CXCL10, and CXCL11 for VL set-points, and found that it exhibited a higher predictive value (84%, *p* = 0.0013) than any chemokine separately (Fig. 6a). These results indicate that higher levels of CXCL9, CXCL10, and the three-chemokine combination panel during PHI are highly predictive of VL set-points. For survival ROC analysis, we divided participants into high and low groups defined by an outcome cutoff of VL > 10^4^ copies/mL. Levels of CXCL10 (> 256 pg/mL; *p* = 0.0198) or the combination of CXCL9, CXCL10, and CXCL11 (≥ 0.5211; *p* = 0.0105) were predictive of VL (Fig. 6b).

As HIV disease progression is determined by both CD4^+^ T-cell count and VL, we next defined the outcome for analysis as a simultaneous VL > 10^4^ copies/mL and CD4^+^ T-cell count ≤ 500 cells/μL. Levels of CXCL10 (*p* = 0.048) exhibited predictive value for HIV disease progression, while CXCL9 (*p* = 0.0786) and CXCL11 (*p* = 0.0605) also showed a similar tendency. Importantly, the three-chemokine combined panel exhibited superior predictive value for HIV disease progression (*p* = 0.0223; Fig. 6c) and the equation, X = 0.007 × CXCL9 + 0.004 × CXCL10 − 0.033 × CXCL11 − 1.724, could be used to provide reference values for HIV disease progression, with X > 0.5211 indicating rapid HIV disease progression (Fig. 6d).

## Discussion

Both determining the exact HIV infection time and obtaining plasma samples from patients with PHI are challenging. In this study, we identified 54 HIV-infected patients with well-documented dates of infection and report comparisons of chemokine and cytokine levels between samples obtained from the same individuals pre-HIV infection and during PHI. During PHI, 16 of 30 chemokines exhibited significant changes post-HIV infection in the same individuals; 12 up-regulated and four down-regulated. We observed that expression levels of the CXC-chemokines, CXCL9, CXCL10, and CXCL11, were dramatically enhanced in plasma, with substantial percentage changes in their levels during PHI. These selective proinflammatory chemokines elicit their biological functions by interacting with a common receptor, CXCR3, a seven-transmembrane GPCR highly expressed by activated lymphocytes, such as CD4^+^ T helper (Th) cells, CD8^+^ cytotoxic T lymphocytes, natural killer (NK) cells, and DCs [27–29]. We also demonstrate that CXCL9, CXCL10, and CXCL11 have potential as novel biomarkers associated with HIV disease progression. Based on our observations, we propose that the combination of CXCL9, CXCL10, and CXCL11 during PHI is a useful biomarker for prediction of HIV disease progression.

Chemokines are crucial players in regulating lymphocyte functions during inflammatory processes [30]. CXCL9, is also induced by IFN-γ in macrophages, implicated in cancer inflammation and viral infections, and participates in T-cell trafficking, chemotaxis, and activation [31–38]. In the present study, the percentage change in plasma CXCL9 was the largest among the chemokines screened. Although levels of CXCL9 are also elevated in oral mucosal, intestinal mucosa, semen, and decidual tissue in vivo and in vitro during HIV infection [39–42], the mechanism by which CXCL9 becomes elevated is unclear. HSV induces CXCL9 expression on human epithelial cells by activating the p38-CCAAT/Enhancer-Binding Protein-β pathway [38], which is a possible mechanism underlying the change in CXCL9 levels during HIV infection. Further, our results show that CXCL9 levels are positively correlated with VL at the sampling point and that patients with high VL (lgVL ≥ 4.5) at the sampling and set-points had higher CXCL9 levels than those with IgVL > 4.5. Elevated CXCL9 is also associated with SIV disease progression and decreased phagocytic activity of mucosal macrophages, which prevents the elimination of bacterial antigens in the small intestine, and induces immune activation [39, 42, 43]. Moreover, blocking CXCL9 can decrease HIV replication in cervical tissues [42, 43]. Additionally, Lajoie et al. [44] found that significantly lower expression of CXCL9 in the genital mucosa was associated with strong protection against HIV infection in HIV-exposed seronegative sex workers. Thus, CXCL9 may be a particularly important factor in relation to VL after HIV infection. Further, during the HIV infection entry process, cortical actin is a physiological barrier to HIV, and HIV uses gp120-CXCR4 signaling to active cortical actin and overcome this restriction [45]. Similarly, CXCL9-CXCR3 signaling may also activate actin to promote HIV entry and post-entry processes, impacting the pace of disease progression. We speculate that CXCL9-induced actin-related signaling may explain its negative function in HIV infection. Moreover, our results are the first to show that levels of CXCL9 correlate negatively with CD4^+^ T-cell count at the 1-year-infection point and we speculate that this may be because, as CXCL9 levels are positively correlated with VL, and VL is negatively correlated with CD4^+^ T-cell count [46], CXCL9 may negatively influence CD4^+^ T-cell count. Furthermore, a study of adults with HIV in Mozambique found that, although levels of some factors were up-regulated during the first month of HIV infection and rapidly decreased in the subsequent months, CXCL9 levels increased and remained high [47]. Thus, the alteration of CXCL9 in the plasma milieu may be a long-term determinant of CD4^+^ T-cell count.

CXCL10, also known as interferon γ-induced protein 10 (IP-10), similar to CXCL9, can be secreted by various cells, including monocytes, leukocytes, endothelia, and epithelia [48], and is up-regulated in numerous diseases, including hepatitis B, tuberculosis, cancer, diabetes, and autoimmune disorders [49–53]. Here, we demonstrated that CXCL10 was elevated during HIV infection and correlated with VL at the sampling point. Additionally, we found that CXCL10 could predict VL at set-point. Some studies have reported that systemic levels of CXCL10 during PHI are positively associated with VL and negatively associated with CD4^+^ T-cell count [22, 54, 55]; however, there have been no previous reports that CXCL10 levels in plasma during PHI can predict VL at set-point. CXCL10 is up-regulated during HIV infection, and may suppress IFN-γ secretion and T and NK cell cytotoxicity [55, 56], potentially partially explaining immune system dysfunction during HIV infection. Cecchinato et al. [57] found that CXCR3^+^ Th cell migration in response to CXCL10 was impaired after HIV infection, and could be rescued by modulating actin polymerization. We speculate that high levels of CXCL10 cause impaired immune cell function, leading to high VL.

CXCL11, also referred to as interferon-inducible T-cell alpha chemoattractant (I-TAC) and interferon-gamma-inducible protein 9 (IP-9), is expressed at high levels in peripheral blood leukocytes, pancreas, and liver [58]. CXCL11 exhibits the highest affinity for CXCR3 among its three selective ligands, followed by CXCL10 and CXCL9 [58]. Our data demonstrate that CXCL11 is significantly up-regulated during PHI, positively correlating with CXCL9 and CXCL10, and negatively associated with CD4^+^ T-cell count at 1-year-infection point. Furthermore, CXCL11 levels can predict CD4^+^ T-cell count, with increased levels detected in mixed cryoglobulinemia, Graves’ disease, and some autoimmune diseases [59–61]. Plasma CXCL11 levels have not been reported in HIV, although higher *CXCL11* mRNA levels were observed in monocyte derived macrophages and dendritic cells which infected with HIV in vitro [62]. Overall, studies of CXCL11 in HIV are limited and the exact mechanism underlying our findings requires further investigation.

As they have a common receptor (CXCR3), CXCL9, CXCL10, and CXCL11 have generally been studied together, and studies of combinations of CXCL9, CXCL10, and CXCL11 in infection, injury, and immunoinflammatory responses have been conducted. Levels of CXCL9 and CXCL10 in lymph nodes are positively associated with disease progression during SIV infection [63]. CXCL9, CXCL10, and CXCL11 can regulate the balance between CD4^+^ effector T-cell subsets and fork-head box P3 (FOXp3)-negative regulatory T cells [64]. Pineda-Tenor et al. [65] found that, in HIV/HCV-co-infected patients, genetic polymorphisms in *CXCL9*, *CXCL10*, and *CXCL11* are associated with sustained virologic response. Recently, the HIV-1 Nef-induced lncRNA, AK006025, was shown to regulate expression of the *CXCL9/10/11* gene cluster in mouse astrocytes [66]; however, plasma levels of CXCL9, CXCL10, and CXCL11 have not been studied simultaneously in HIV-infected individuals. Our results demonstrate that a combined panel of CXCL9, CXCL10, and CXCL11 has predictive value for HIV disease progression, when both CD4^+^ T-cell count and VL are considered. Testing for plasma chemokine levels is convenient and simple; therefore, our model has potential value for clinical application to predict HIV disease progression.

## Conclusion

Our study highlights the roles of CXCL9, CXCL10, and CXCL11 in viral control and disease progression during HIV infection. In addition, interactions and dynamic changes among these three chemokines may provide important information regarding the mechanisms underlying their roles during PHI. Overall, our results indicate that the combined panel of CXCL9, CXCL10, and CXCL11 can predict HIV disease progression using the formula, X = 0.007 × CXCL9 + 0.004 × CXCL10 − 0.033 × CXCL11 − 1.724, and threshold value of 0.5211.

## Data Availability

Authors can confirm that all relevant data and materials are available on request from the authors.

## Abbreviations

HIV: human immunodeficiency virus
PHI: primary HIV infection
MSM: men who have sex with men
VL: viral load
Lg VL: log_10_ VL
HSV: herpes simplex virus
SIV: simian immunodeficiency virus
HCV: hepatitis C virus
CXCL9: C-X-C motif ligand 9
CXCL10: C-X-C motif ligand 10
CXCL11: C-X-C motif ligand 11
GPCRs: G protein-coupled receptors
CXCR3: C-X-C motif receptor 3
DCs: dendritic cells
Th cells: T helper cells
NK cells: natural killer cells
IP-9: interferon-gamma-inducible protein 9
IP-10: interferon-gamma-inducible protein 10
I-TAC: interferon-inducible T-cell alpha chemoattractant
lncRNA: long non-coding RNA
